# 743 Virtual burn care - friend or foe? A systematic review

**DOI:** 10.1093/jbcr/irac012.296

**Published:** 2022-03-23

**Authors:** Claudia Malic, Eli Mondor, Jaymie Barnabe, Robyn M Laguan

**Affiliations:** Children's Hospital of Eastern Ontario, Ottawa, Ontario; Carleton University, Ottawa, Ontario; Carleton University, Ottawa, Ontario; The Ottawa Hospital, Ottawa, Ontario

## Abstract

**Introduction:**

Interest in virtual care has grown, but evidence surrounding its use for burn injuries is variable. This systematic review assesses the impact of virtual burn care in the past decade (2010-2020) by providing an overview of recent advances in the field. Data on efficacy, feasibility, cost-effectiveness, usability, pros/cons, satisfaction/acceptability, clinical outcomes, and triage effects are presented. Conclusions on its post-pandemic sustainability are drawn.

**Methods:**

A systematic review with qualitative synthesis was performed according to PRISMA guidelines. Quality of included studies was assessed by validated tools. CINAHL, OVID MEDLINE, APA PsycINFO, and the CENTRAL trials registry were searched. Grey literature was searched for in OAIster, Duck Duck Go, Bandolier Knowledge, LILACS and McMaster Health Systems Evidence. Primary literature published between 01/01/2010-12/31/2020 investigating any of the noted outcomes of interest was retrieved for data extraction.

**Results:**

A total of 486 studies were identified for screening. 412 and 26 citations were excluded in title/abstract and full text screening, respectively. After removing 8 unretrievable works and 3 straggling duplicates, 50 citations were included. Most works were published from 2016-2020 (n=35, 70%). The most common uses (with some overlap) were acute assessment (n=35, 70%), remote follow-up (n=18, 36%) and tele-rounding (n=4, 8%). Remote photographic burn size (not depth) estimation was found feasible and acceptably accurate. Patient and provider satisfaction was high overall. Patient outcomes with virtual follow-ups were largely comparable to equivalent in-person services, though some adjunct programs saw little benefit. Increased specialist access, more accurate assessment/triage and saved travel time/cost were commonly noted. Challenges included logistics and language barriers for international interventions, IT issues and internet access limitations, HIPAA compliance and some wound/scar assessment challenges (e.g. burn depth and scar vascularity).

**Conclusions:**

Evidence suggests that virtual burn care is largely safe, efficacious and could be feasible for continued use post-COVID-19 provided technological infrastructure is attainable and suitable regulation exists. Virtual acute specialist burn assessment is particularly well supported.

